# Continuous and binary sets of responses differ in the field

**DOI:** 10.1038/s41598-022-17907-4

**Published:** 2022-08-23

**Authors:** Noelia Rivera-Garrido, M. P. Ramos-Sosa, Michela Accerenzi, Pablo Brañas-Garza

**Affiliations:** 1 Economics, LoyolaBehLab, Universidad Loyola Andalucía, Seville, Spain; 2 Fundación ETEA, LoyolaBehLab, Universidad Loyola Andalucía, Seville, Spain

**Keywords:** Psychology, Human behaviour

## Abstract

This paper conducts a pre-registered study aimed to compare binary and continuous set of responses in survey questionnaires. Binary responses consist of two possible opposing response options (Yes/No). Continuous responses are numerical, where respondents can indicate an option on a 0–10 horizontal blind line. We study whether feasible sets of binary and continuous responses yield the same outcome (distribution) and have the same cost (duration in minutes). We collect data from 360 households in Honduras that were randomly assigned to Yes/No questions or given a slider (0–10 visual scale) to mark their responses, therefore, we provide *causal evidence*. We find that respondents are 13% more likely to respond “Yes” and spend 2.1 min less in the binary setting. Additionally, the results suggest that the type of question matters.

## Introduction

The literature on survey design, and more specifically the choice of response format, offers a wide typology of questions to evaluate the degree of agreement with an issue^1–5^. Agree-disagree scales are used to obtain the degree of agreement with an issue^6^. However, item-specific questions (in which responses specifically refer to the question) are recommended when the scale options in the questionnaire refer directly to the issue under evaluation^7,8^. On the other hand, perception questions are used when the interviewer’s goal is to obtain information on how respondents perceive certain issues. This type of question can be less problematic when the response reveals personal information^9^. Perception questions may induce social desirability bias and can present a reliability problem with open questions, as the wording used might have a different meaning for respondents^10^. In this case, the use of closed-ended questions and also to know the realities that respondents have to report are recommended^11^.

In social sciences, one of the most widely used types of closed questions is dichotomous scales (binary responses) in which two opposing responses are provided (typically “Yes” or “No”). This typology is generally used when the goal is to evaluate the direction of the answer (agreeing or disagreeing with an issue). There is considerable consensus on the meanings of the options^12^ and they are easy for the respondents to understand. They also involve low costs and less time for both researchers and respondents^13^. In addition, it has been shown that people correctly answer factual questions (content-specific questions that require only one correct answer) more often when the correct answer is “Yes”^3^. This format also requires less interpretative efforts compared to longer questions^12^, although the lack of neutral response may alter the results. For example, it may lead to acquiescence bias, which refers to a respondent’s tendency to agree with a statement without considering the content of the item or to please the researcher^3,14–16^. Moreover, this format could be problematic for respondents with neutral attitudes due to the lack of accurate mapping between moderate responses and the dichotomous options offered. As a result, such a format would require a major cognitive effort^12,17,18^.

Continuous rating scales enable researchers to obtain the exact degree of agreement with an issue. When continuous rating scales are numerical, respondents can indicate an option on a horizontal line by marking precise numbers that express the degree of agreement with an issue with respect to a maximum number, usually 10 or 100. The use of this metric scale allows subjects to easily transform their opinions, whether extreme or mild, into replies, thus reducing distortion^19^. Nevertheless, point meanings may become less clear as the respondent cannot differentiate between one number and another^12^.

When choosing between binary and continuous rating scales, it is important to evaluate the advantages and disadvantages of each type of question. In terms of statistical evaluations, continuous scales allow for a wider range of procedures^20^. Regarding response time and complexity, continuous scales require more time to explain the set of possible answers and to think about their conversion into numbers, which implies higher reasoning costs^13,19^. However, compared to binary responses, a continuous set of feasible answers provides better quality of information^12^. In binary scales, where respondents have to convert mild responses into Yes–No answers, respondents are forced to make computations to transform their opinion in the middle to an extreme answer, resulting in an inaccurate choice, which consequently may manifest a higher degree of agreement that would not be reached if middle responses were available (i.e., acquiescence or satisficing^3,16^). Moreover, binary scales do not allow for nuanced responses or permit studying respondents’ expectations at the individual level. Therefore, if respondents have problems computing their mild thoughts into extreme responses, biases arise.

This study investigates the extent to which the use of binary and continuous scales yields different results. Specifically, we examine if the probability of agreeing with a statement is the same when binary and continuous sets of responses are used and subjects have strong opinions regarding the content of the survey, in our case: sexual and reproductive health (see “Methods”). To do so, we conduct a field experiment in Honduras with 360 randomly selected participants. Half of the participants were randomly assigned to the Yes-No binary treatment while the other half were assigned to the continuous treatment which included the same questions but a different set of responses. Specifically, subjects in the continuous treatment had to indicate the degree of agreement with a statement on a scale of 0–10 using a slider.

How continuous answers are captured is also important. In this study, we use sliders, a visual analog scale that allows respondents to mark their responses in an interval of 0–10. Sliders are more engaging than other visual scales^21,22^, and increase the time and effort needed to provide an answer^18^. Sliders also allow respondents to communicate exact values and simultaneously convey to the respondent how precise the expected answer should be^23^. However, the use of sliders to collect responses can be time-consuming^13,19^. In addition to longer completion times, there are other challenges, such as how to handle the starting position of the marker^20,24^, or the inclusion or not of anchors and labels^20,25^. To overcome these difficulties, we used partially labeled sliders with a scale range of 0-to-10 points that only indicate the extreme points (0 and 10). Additionally, the experiment was conducted by enumerators who read out and explained the instructions to participants. For the continuous questions, they were also in charge of explaining their correct use to the respondents and avoiding issues with incomplete data or with the slider bar movement and interpretation^24^, therefore, removing all the above-mentioned difficulties.

To compare responses between treatments, we discretize the continuous answers. We assign the binary option “No” if the selected response is below 5. Respondents who indicate agreement of more than 5 points are assigned to the binary option “Yes”. When the indicated option is 5, the answer is randomly assigned to “Yes” or “No”. Additionally, we study whether the wording of the questions, such as the use of negative words (e.g., no, nothing) or prescriptive expressions (e.g., act as, take care of, restrict activities) also affects the probability of agreement. We also analyze the differences in terms of the response time for both treatments.

We find that respondents are more likely to respond “Yes” in the binary setting compared to the continuous setting. In particular, the probability of responding “Yes” is 2.3 times higher for the binary than the continuous questions. Regarding response time, binary questionnaires took 2.1 min less, which implies an average reduction of 42.5% with respect to the continuous questionnaires. Our results hold when excluding males from the sample and subjects with lower cognitive abilities. Given the content of the questionnaire we extend the analysis to the wording of the questions. The literature has shown that question-wording also plays an important role in obtaining accurate responses. Negatively worded questions have been defined as those in which disagreement would be a good answer^15^. Negatively worded questions are usually included in surveys to avoid fatigue throughout the questionnaire, acquiescence^15,26,27^ and to prevent respondents from choosing the same answer^28^. Nevertheless, one of the issues that arises with negative questions is the additional processing effort they require, particularly in binary sets, where respondents have to decide to agree or disagree with a question that includes negation, which may generate confusion^15,29,30^ (see also^31^). In the same line, our data show that when negative wording is used in the questions, the probability of agreement jumps to 37% in binary sets compared to continuous ones.

## Methods

### Protocol

In certain contexts, asking people to reveal their opinion is challenging. For instance, voters might have concerns about revealing their (private) vote in pre-election polls. Similarly, citizens might also have concerns about sharing private information with the government when they are likely to be assigned to a public program. In both cases, participants might consider that their responses matter and therefore they might feel obliged to complete the survey consciously. To check whether there are differences between binary and continuous sets of questions, we select a topic on which subjects have very strong opinions: sexual and reproductive health.

In May 2019, we ran the experiment in Santa Rosa de Copán (Honduras); an area characterized by high rates of teenage mothers, with a rate of 28.17% of teen pregnancy (mothers of 9–19 years old)^32^. In fact, according to^33^, a Honduran adolescent who is completely “illiterate” about sexual and reproductive health is 44% more likely to become pregnant than a fully informed adolescent. Given the general sensitivity surrounding this topic particularly in this region, we decided that this sample would be ideal to test the impact of the question setting on responses. Considering that the onset of menarche occurs between the age of 9 and 16 years, the eligibility criterion to be part of the sample was having a child between 6 and 9 years old. This was motivated by the fact that children need to have access to information about the menstrual cycle and sexual and reproductive health before menarche, which is an essential part of menstrual health^34^. The experiment was conducted by a consultancy firm (PILARH) in four districts in which 360 households were randomly selected. Following local procedures, PILARH contacted the territorial Office of the Education Secretary which permitted us access to eleven schools representing different socioeconomic levels. The selected schools were: Divina Providencia, Jerónimo J. Reina, Jesús Banegas Membreño, Jorge Portillo, José Cecilio del Valle, José María Medina, Los Ángeles, Manuel Bonilla, San Antonio, San José and Santo Domingo Savio. School representatives talked to parents and asked them to participate in the study. Only one adult per household was asked to voluntarily complete the questionnaire. The entire experiment consisted of four experimental tasks and a questionnaire, which was completed following the tasks. This study is entirely based on the questionnaire.

The experiment was conducted by twelve enumerators. They were specifically trained for the field work and were in charge of reading and explaining the instructions to the participants. Each of the enumerators received a list of households to visit, which were randomly allocated to the treatments before the visit. The enumerators had no influence on the selection of households to ensure that none of them conducted all the questionnaires for households belonging to the same school. All the interviews were conducted face-to-face in the participants’ households. The questionnaire was paper-based and included 30 questions divided into 6 blocks (A–F) of 5 items each about menstruation and reproductive rights. The original questionnaire in Spanish and its translated version in English can be found in sections S1 and S2, respectively, of the Supplementary Information.

### Ethics and pre-registration

This study was approved by the Ethics Committee of Universidad Loyola Andalucía and was conducted according to the principles expressed in the Declaration of Helsinki. All participants signed informed consent before participating. The field study was pre-registered on April 15, 2019 and made public on April 25, 2019 at https://aspredicted.org/ps766.pdf.

### Design

To assess the effect of binary versus continuous sets of responses in a questionnaire, subjects were randomly allocated to $2\times 2$ treatments (between-subjects): $\{B, C\}$ $\times$ $\{Order1, Order2\}$.

**Figure 1:**
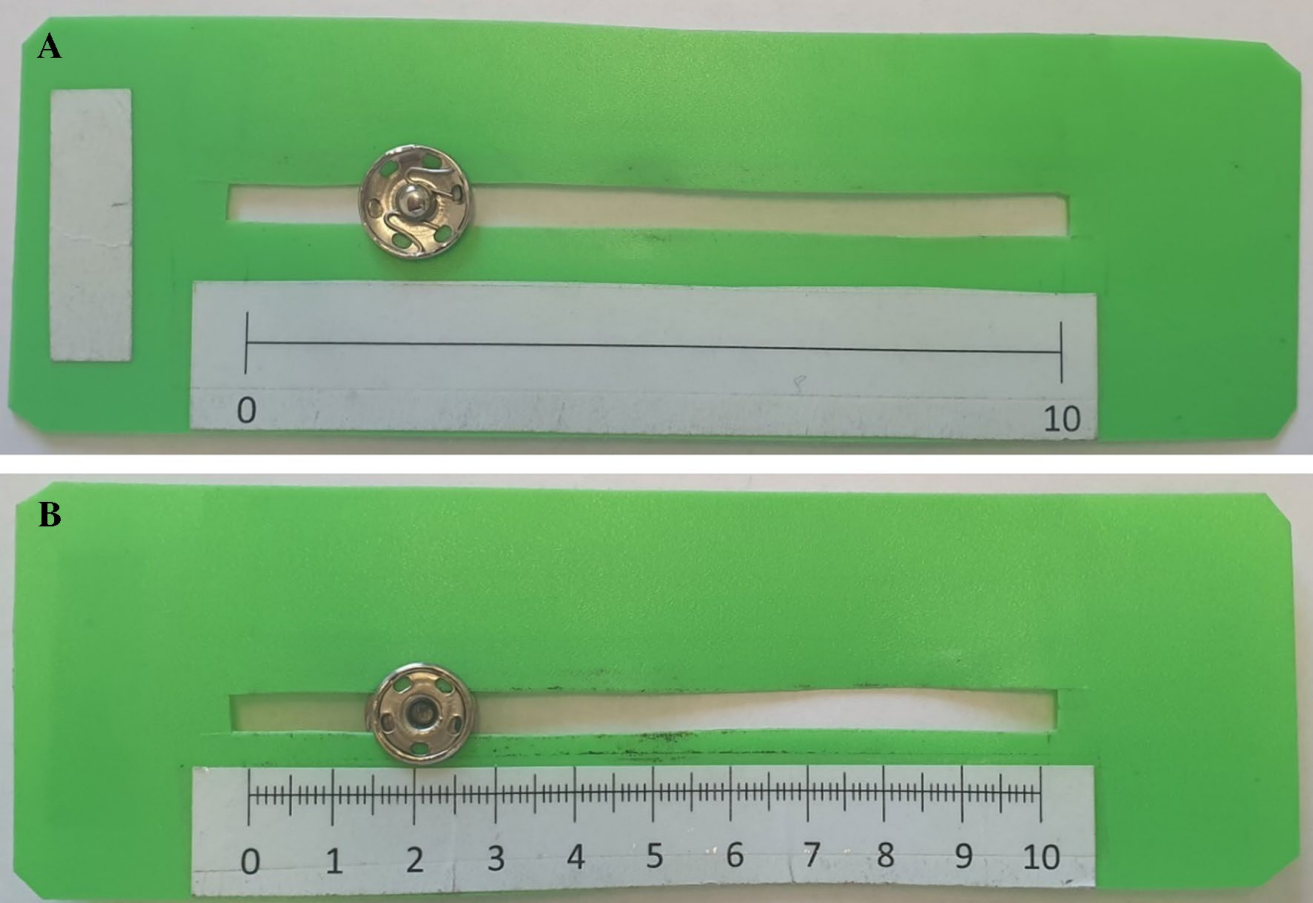
Slider. (**A**) Respondents view of the slider (front side). (**B**) Enumerator view of the slider (back side).

- {B, C} refers to binary and continuous sets of responses, respectively. The binary set of responses corresponds to the case in which the set of possible answers is limited to *“Yes”* or *“No”* (i.e., dichotomous question). This information was provided to the respondents by the enumerators. In contrast, the continuous set of responses allowed subjects to indicate an option within a given interval. Specifically, the enumerators captured respondents’ answers using partially labeled sliders on a scale of 0–10 points that only indicated the extreme points (0 and 10). In this case, enumerators told the respondents to use the slider for each question (Fig. 1) and that 0 indicated “completely sure it will not happen” and 10 “completely sure it will happen”. Respondents could drag the marker and drop it at a desired location on the horizontal line. The starting position of the marker was 5. Respondents were able to see the front side of the slider (panel A), while the enumerator could observe the back side (panel B).
- {Order1, Order2} refers to the order in which the 30 questions (organized in 6 blocks of 5 items each) were presented. Specifically, 52% of the participants started with blocks A, B, and C (and then D, E, and F) while the remaining 48% started with blocks D, E, and F, and then completed blocks A, B, and C. The order of the items within each block remained the same.

The enumerators received instructions about the treatment and the order of the questions to be used for each household.

We were also interested in comparing the time spent when using the binary instead of the continuous set of responses. To that end, we recorded the time (in minutes and seconds) each participant took to complete the entire questionnaire (given the paper-based nature of the questionnaire, we could only account for the time required to complete the whole questionnaire, but not each individual question). Finally, to account for respondents’ ability, we used an extension of^35^ developed by^36^. This test consisted of several questions that allowed us to determine whether the respondents were able to deal with probabilities (see Supplementary Information S3).

### Sample

The total sample consisted of 360 subjects. However, seven of them did not answer all the questions, resulting in a response rate of 98%. Supplementary Table S1 presents the descriptive statistics of the key variables in our analysis. All the subjects surveyed were residents in Honduras at the time of the survey and had a child between 6 and 9 years old in one of the participating schools (see Supplementary Table S2). The average age of the participants was 34 years and they were mostly women (87%) with secondary or less than secondary education. This over-representation of women may be because responding to the questionnaire was voluntary and, according to PILARH, it is usually women who participate in school-related activities and dedicate more time to children’s care. Most respondents have a daughter living at home (65%). Most of the individuals were not of a particular ethnicity (70%), and 29% was low-ability. In addition, 77% of the individuals in the sample indicated that they had enough money to feed their children.

All the subjects completed the same questionnaire with only one difference, the type of answer (binary vs continuous). Of the participants, 52% answered binary questions (Yes/No), and 48% answered continuous questions.

Finally, we provide the balance of the randomization across treatments in our experiment (see Supplementary Table S1, column 6). Our sample is balanced in all the individual and other survey characteristics (age, ethnicity, sufficient income, ability, order of questions, and education) except gender ($p=0.008$) and marginal differences in secondary education.

### Standardization

To determine whether individuals respond differently to the same question depending on whether it is asked in binary form (*Yes/No*, where *Yes* is *1* and *No* is *0*) or continuous form (scale from 0 to 10, from disagreeing to agree), the responses to the two types of questionnaires (binary and continuous) need to be comparable. Following the existing literature^37,38^, we discretize the continuous responses and then compare them with the binary responses. Note that respondents have no information on the codification of their answers.

Let $r\in [0,10]$ be the set of feasible answers for the continuous questionnaire. We generate the following binary variable, *d*.

$$\begin{aligned} d={\left\{ \begin{array}{ll} 0, &{} \text {if r<5} \\ 1, &{} \text {if r>5} \\ 0, &{} \text {with prob=0.5 if r=5 } \\ 1, &{} \text {with prob=0.5 if r=5 } \end{array}\right. } \end{aligned}$$

If the individual claims to agree with a statement by indicating more than 5 ($r>5$), the binary equivalent is “Yes” and it is assigned to 1. If the response is less than 5 ($r<5$), the equivalent is “No”, and it is assigned to 0. Finally, if the individual indicates a neutral response of 5 ($r=5$), we randomly assign the answer to 0 or 1.

We opted to split answers equal to 5 as this value represents the mean point in the continuous set. Using the slider, we can measure the intensity of an opinion. Similarly to our case, the well-known Likert scale questions^39^ also measure this intensity. In the original 5-point Likert scale questions the mean value indicates “neither agree nor disagree”, values above the mean indicate agreement with the statement, and values below the mean indicate disagreement with the statement^40^. Following similar reasoning, we assume that: (1) responses greater than 5 indicate agreement with the survey question; (2) responses less than 5 indicate disagreement with the survey question; and (3) responses equal to 5 indicate neither agree nor disagree. Then, as a result, when the response is 5, we randomly assign the individual to “Yes” or “No”.

## Results I: Aggregate data

**Figure 2:**
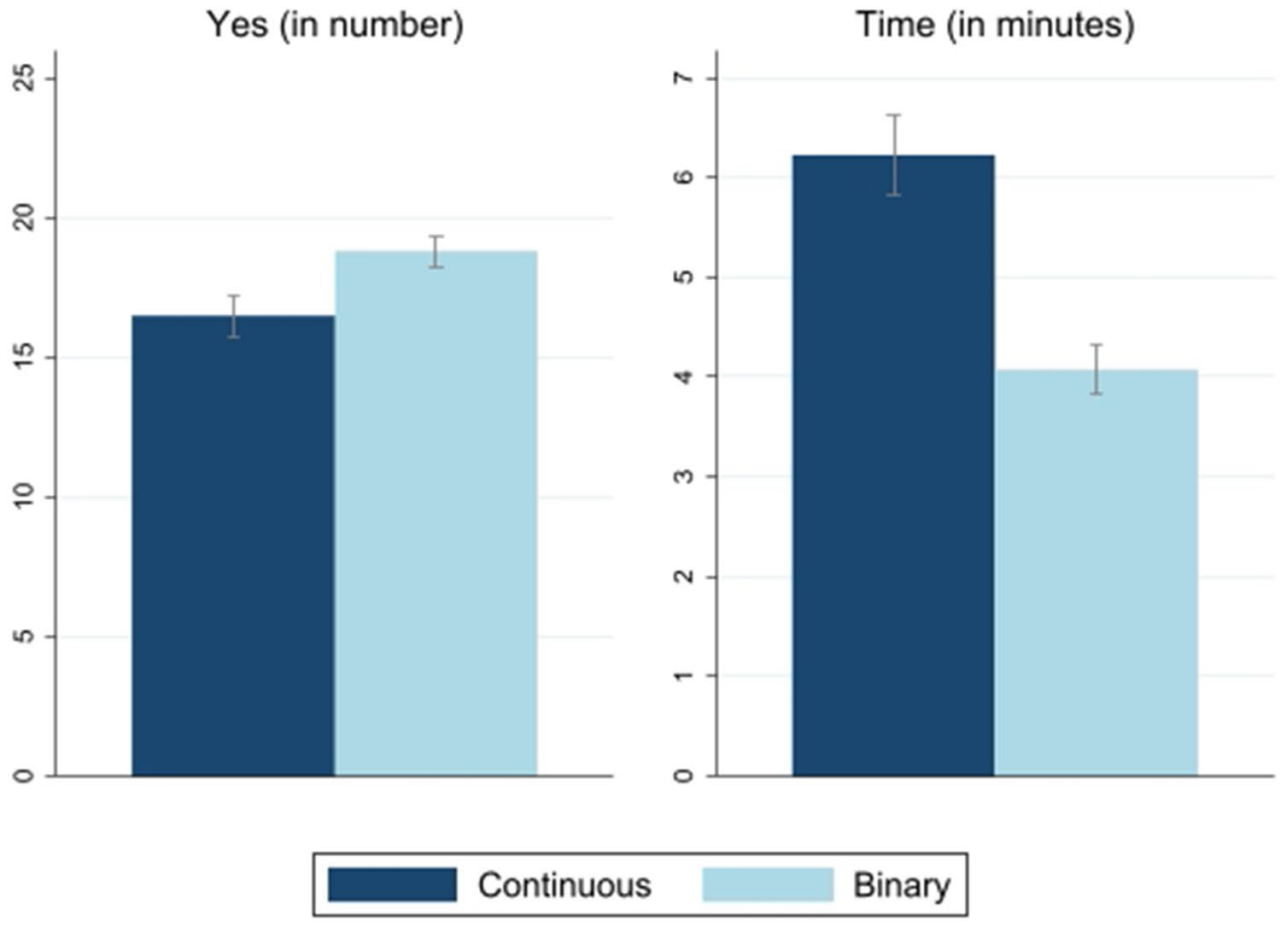
Binary vs. continuous settings. Left: average number of times the subjects agree in the questionnaire. Right: response time in minutes. Lines with caps represent 95% CI. Sample: all.

We start by showing the results of the participants’ behavior along with the entire experiment. Recall that all participants replied to 30 questions in random order (52% starting from block *A*, and 48% starting from block *E*). Figure 2 shows the average number of *“Yes”* responses, along with the average time (in minutes) respondents spent on the entire questionnaire in both continuous and binary forms.

We observe that the probability of agreement with the survey question is higher when the question is binary, and that the average time is lower in the binary setting. Table 1 shows the regression analysis. In addition, the difference between the binary and the continuous questions is statistically significant at the 1% level (Table 1).

It is important to recall that the subjects were randomly assigned to treatments. Hence our results imply *causality*. Therefore, we conclude that:

1. Binary settings produce higher shares of *“Yes”* responses.
2. Continuous settings are more time-consuming.

Before examining the data question by question, we want to explore whether Result 1 and Result 2 are robust to certain variations. First, we consider only women in the sample (87%) and repeat the same exercise to check whether the results are driven by the very few men in the sample (13%). Second, we remove subjects facing problems with probabilities (see Table 1).

**Table 1:** Binary vs. continuous set of responses (aggregate level).

Outcome:	Aggregate Number of “Yes”			Time (min)		
Sample:	All	Women	Intermediate/high ability	All	Women	Intermediate/high ability
Binary	2.317*** (0.322)	2.253*** (0.388)	2.534*** (0.392)	− 2.159*** (0.155)	− 1.991*** (0.230)	− 2.486*** (0.172)
Dep. Var (Mean)	17.66	17.72	17.56	5.11	4.97	5.18
Observations	353	306	249	338	295	242
R-squared	0.069	0.068	0.082	0.201	0.218	0.243

The dependent variable Aggregate Number of “Yes” responses is the number of “Yes” responses by a subject in the questionnaire. The dependent variable Time, is the duration of the questionnaire (in min). The table reports the estimated coefficients for a dummy variable equal to one if the set of responses is binary. Note that the number of observations is lower when the dependent variable is time because in some cases we have no information on the duration of the survey.

Robust standard error in parenthesis. Standard errors clustered at the school level.

***p < 0.01, **p < 0.05, *p < 0.1

**Figure 3:**
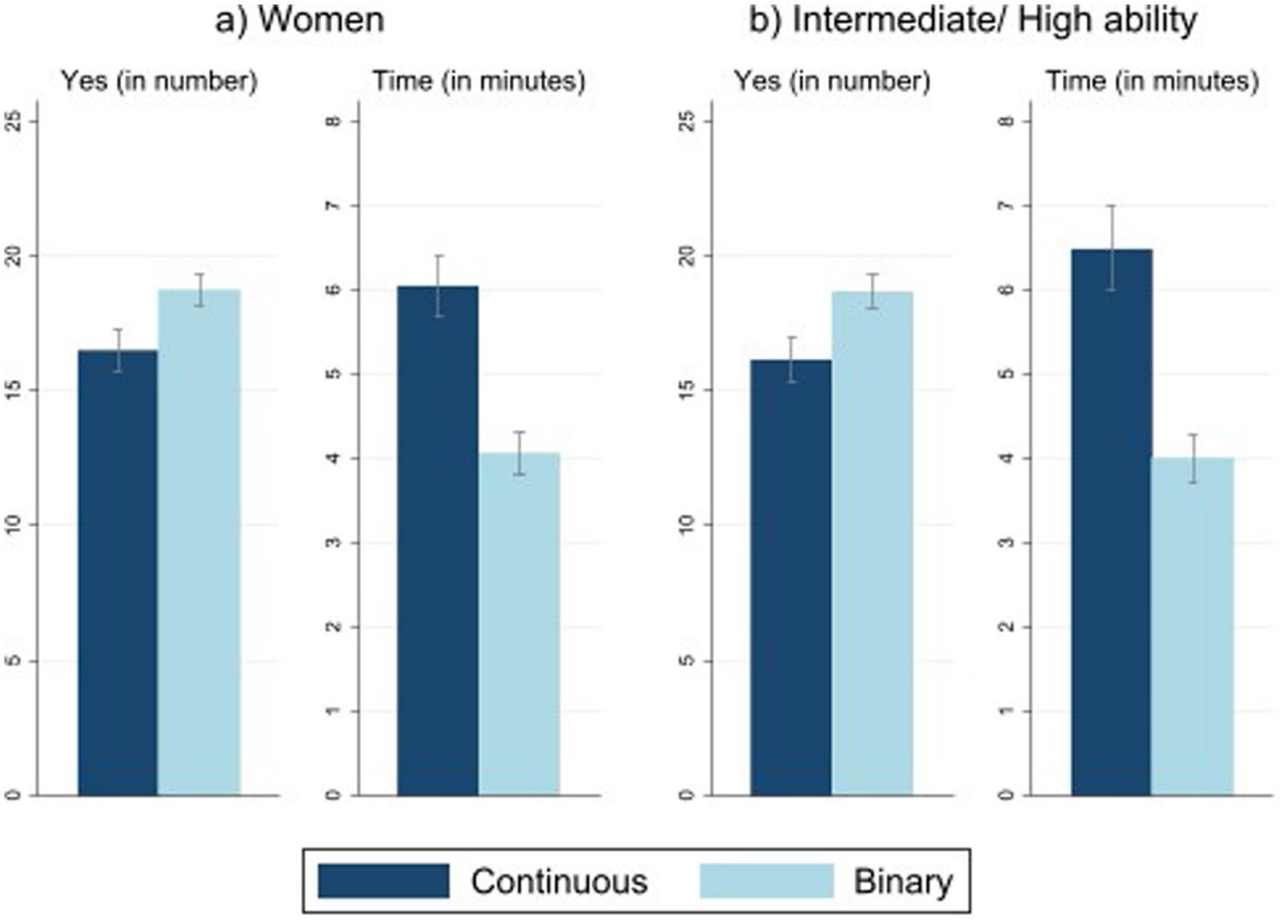
Binary vs. continuous settings. Left: average number of times the subjects agree in the questionnaire. Right: response time in minutes. (**a**) Women, (**b**) Intermediate/high ability. Lines with caps represent 95% CI.

Figure 3a repeats the previous Fig. 2 using the sample restricted to women only. The results are similar to the whole sample. Therefore, our results are not driven by men and *Results 1 and 2 are confirmed for women.*

Now, we focus on respondents’ cognitive abilities. We want to see whether Result 1 and Result 2 hold once we consider that subjects may face problems when they are asked about probabilities and expectations.

Figure 3b reflects the same as Fig. 2 for individuals who show intermediate or high-ability according to the extension of^35^ developed by^36^. Thus, we conclude that *Results 1 and 2 are not driven by the respondents’ cognitive abilities*.

In conclusion, the use of binary (instead of continuous) sets of responses causes subjects to respond *“Yes”* more often (or *“No”* less often) and complete the survey much faster. These results are robust to excluding men and low-ability respondents. Supplementary Table S3 displays the results when controlling for different characteristics (age, gender, ethnicity, school, sufficient income, having a daughter, education, and the order of the questions in the survey). The results hold. Supplementary Table S4 shows the results when including interactions by gender and respondents’ abilities. We find no gender differences and marginal differences for intermediate/high-ability.

## Results II: Question by question

**Figure 4:**
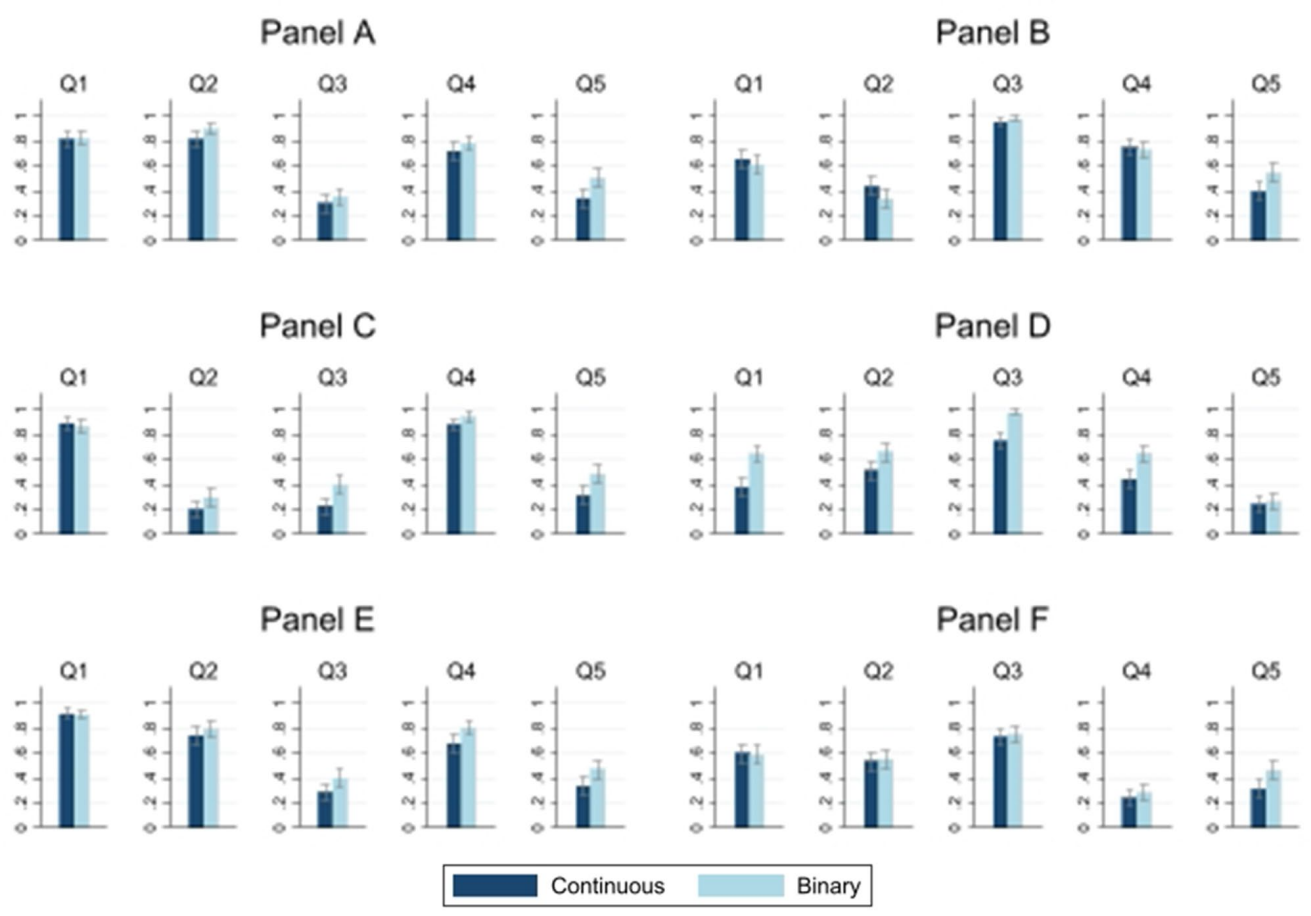
Binary vs. continuous settings: probability of agreement. Item by item. Lines with caps represent 95% CI.

Figure 4 shows the average number of *“Yes”* (and *“No”*) responses to each question. As can be seen, Result 1 (higher number of *“Yes”* responses to binary questions) does not hold for every single question. Indeed, we see that many items are almost identical in the binary or continuous setting. In particular, we find that 10 out of 30 questions are different and, most importantly, the direction of the difference is always the same (see Supplementary Table S5, $p<0.05$). That is, from the ten questions that report different numbers of *“Yes”* responses, all of them show a higher probability of agreeing with the survey question when it is in binary rather than continuous form. This difference is statistically significant at the 5% level. Similar results were found using the Bonferroni correction method (see Supplementary Table S5).

Result 3: In 33% of cases there are differences between the binary and continuous settings. In the other cases, the differences are always in the same direction (positive) as in Result 1.

Supplementary Fig. S1 shows the distribution of responses to each question in the continuous setting. Interestingly, substantial heterogeneity was found between questions. This indicates that, first, subjects do not share similar values. Second, and most importantly, the observed responses are widely spread across the intervals, with the focal point (number 5) not being particularly salient. This latter finding might be indicating that individuals have strong opinions about the questions.

For sake of completeness we repeat the analysis for women only and for the sub-sample of subjects who passed the cognitive test. Results remain in the same direction (Supplementary Table S6).

Our results also hold when we remove individuals who give the neutral response 5 (see Supplementary Table S7) and when we control for individual and other survey characteristics (See Supplementary Table S8).

### The role of wording

Next, we explore the role of question-wording in explaining Result 3. To do this, we distinguish three types of survey questions (SQ henceforth): negative, prescriptive, and other. Similar to the previous literature, *Negative* refers to questions which include words like “no” or “not”^15^.

*Prescriptive* refers to those questions that include wording like “have to” or “must be”, or refer to what one should do, what is right or wrong, or good or bad^41^. *Other* questions refer to questions that cannot be classified as either negative or prescriptive. SQ5 in Panel A; SQ5 in Panel B; SQ5 in Panel C; SQ1, SQ4 and SQ5 in Panel D; SQ5 in Panel E, and SQ5 in Panel F are negative questions. SQ2 to SQ4 in Panel B are prescriptive questions.

First, we analyze differences at the aggregate level in the binary and continuous settings for each group of questions (negative, prescriptive, and other). That is, we compare the total number of “Yes” responses in binary and continuous forms. We start with the whole sample. Table 2 reports the average difference in the number of “Yes” responses to binary rather than continuous questions for each group of questions. Regarding the group of negative questions, those who completed the binary questionnaires indicated “Yes” 1.3 times (or 36.8%) more than those who completed the continuous questionnaires. The average number of “Yes” responses is also higher in the binary questionnaire when considering the group of other questions (1.15 times or 9.5% higher). The difference is not statistically significant when considering the group of prescriptive questions.

**Table 2:** The role of question-wording.

	Negative	Prescriptive	Others
**Sample: all**			
Binary	1.258*** (0.230)	− 0.0985 (0.0958)	1.157*** (0.296)
Dep. Var (mean)	3.42	2.09	12.14
Observations	353	353	353
R-squared	0.076	0.004	0.036
**Sample: women**			
Binary	1.128*** (0.251)	− 0.126 (0.0921)	1.251*** (0.331)
Dep. Var (Mean)	3.44	2.0	12.18
Observations	306	306	306
R-squared	0.062	0.008	0.045
**Sample: intermediate/high-ability**			
Binary	1.363*** (0.221)	− 0.0890 (0.0969)	1.260*** (0.317)
Dep. Var (Mean)	3.29	2.08	12.1
Observations	249	249	249
R-squared	0.093	0.004	0.043

The dependent variable is the number of “Yes” responses by subjects in the questionnaire. The “Negative” column refers to the negative questions, the “Prescriptive” column refers to the prescriptive questions, and the “Others” column refers to questions that cannot be classified as either negative or prescriptive.

Robust standard error in parentheses. Standard errors clustered at the school level.

***p < 0.01, **p < 0.05, *p < 0.1.

Result 4: The probability of agreeing with the survey question is higher when a binary set of responses and negative wording are used (36.8%).

We repeat our analysis for women and removing subjects with low-ability according to the results obtained from^35^ (Table 2) and controlling for individual and other survey characteristics (see Supplementary Table S9). Supplementary Table S10 also replicates the results running a regression with explanatory variables that include a dummy for the treatment (binary responses), dummies for each type of question (negative and prescriptive), and interaction terms between the dummies and the treatment. Result 4 is also confirmed.

## Discussion

We investigate how responses change when a binary Yes-No question format or a continuous rating scale is used. Using data from 360 households in Honduras this paper provides *causal evidence* of differences in the probability of agreement and the response time of the questionnaire. We also provide non-causal evidence of the impact of negative wording. We compare binary and continuous responses by discretizing the continuous variable.

Our first result shows that, on average, the probability of agreement is 13% higher in binary sets compared to continuous sets. The fact that the binary setting yields higher agreement rates is not new. Indeed, this type of survey question is affected by acquiescence, that is, the tendency to agree with the question without considering the content of the item (see^3,16^).

Second, we measure response times (length of the interviews in minutes) and, as expected, Result 2 shows that continuous settings require more time than binary settings. Specifically, opting for Yes-No responses reduced the average length of the survey by 2.1 min, which implies a reduction of 42%. This result is also expected given the wider range of options offered to the respondents, who may need more time to accurately map their opinion in an interval of 0–10.

To check whether Results 1 and 2 are robust, we analyze the probability of agreement focusing on two particular sub-samples: women and respondents’ ability. When we consider only women in the sample, which account for 87% of the sample, we find similar results: the probability of agreement is 2.25 times higher in the binary than in the continuous sets. The length of the questionnaire is also 1.9 min shorter when using binary questions for this sub-sample. Hence, the differences in the probability of agreement and time to complete the questionnaire between binary and continuous sets of responses are not driven by men.

Additionally, we consider respondents’ ability using an extension of^35^ (see also^36^). We find that 71% of the sample responded correctly to these questions. Using this sub-sample, we test our data and find similar results: high-ability respondents require on average 2.49 min less when using a binary rather than a continuous questionnaire, which implies a reduction of 48%. We also find that the probability of agreement is higher in binary sets compared to continuous sets. Specifically, respondents answer “Yes” 2.5 times more (or 14.4%) in binary sets^3,16^.

Third, we also estimate the differences in the responses for each question. Result 3 shows that 33% of all questions present significant differences between the binary and the continuous sets, with a higher agreement in the binary setting in all cases. These results hold when we only consider women and when we take the high-ability sub-sample of respondents.

To go a step further, we analyze whether the wording of the questions matters. In particular, we distinguish between *negatively* worded questions, that include negative adverbs in the sentence. Specifically, 8 out of 30 questions include negative words, of which 7 show significant differences when we compare the binary and the continuous treatment. This specific group of questions shows 37% more agreement in the binary than in the continuous sets. The group of questions classified as *“other”* also shows 9.5% more agreement in the binary than in the continuous sets. However, the group of questions with *prescriptive* words shows no differences. The use of negatively worded questions is recommended to avoid acquiescence and inattention. However, we obtain the opposite result. Therefore, we advise the survey researcher to use negatively worded questions with caution as its use might cause reliability and validity problems, overall in binary sets and with individuals with lower general education (see also^26^).

Although the results indicate that binary questions can be responded more quickly and are thus more efficient in terms of time and cost, one of the limitations of this research is that we do not know the true answers: the information we obtained from the questionnaire does not allow us to assess whether binary or continuous settings reflect true answers^42^.

In short, in the absence of true values we cannot prove which setting is more accurate. Furthermore, it is also important to remark that the sensitivity of the topic and the use of perception questions to avoid possible situations of social desirability bias might have an impact on subjects’ responses^9,43,44^ as respondents may have problems in disentangling perception versus preferences. An interesting line of further research would be to compare both settings when the social norm is clearly defined.

While our results are remarkable, it should also be noted that our study has two major limitations that preclude us from generalizing the results. First, our sample is very specific: inhabitants of semi-rural areas of Honduras (mostly women). Second, the topic—sexual and reproductive health—is fairly sensitive. Further research with a more general population and less sensitive questions would be necessary.

## Concluding remarks

This paper aimed to test whether using binary or continuous sets of questions leads to different responses. To this end, we designed a quasi-experimental survey where subjects were randomly assigned to the binary or continuous treatment, hence we have provided causal evidence. The experiment was conducted in Honduras with 360 randomly selected participants. At the aggregate level, we found that subjects are more likely to respond “Yes” in the binary setting (Result 1). We also found that subjects in the binary treatment spent 42% less time responding to the entire questionnaire (Result 2). Thus, using a continuous set of responses comes at a higher cost. We also tested whether both results still hold when the sample is composed only of women (87% of the sample) and the results are similar. We also tested whether the results remain constant when controlling for cognitive abilities. The proportion of subjects classified as having low-ability is 29%. The results obtained when removing this subgroup of participants are similar to those using the whole sample. We then estimated the difference between the binary and the continuous treatment question by question and found two relevant results. First, the differences between binary and continuous sets do not occur in every single question but only in 33% of them. However, the difference between a binary and continuous sets always goes in the same direction. That is, the probability of agreeing with the survey question is higher when using a binary instead of a continuous set of responses. Finally, we analyzed the role of question-wording. To that end, we differentiated between questions with negative, prescriptive, and other wording. We found that negative wording increases the probability of subjects responding “Yes” by 37%. No differences were found between binary and continuous questions when using prescriptive wording; in sum, our paper provides causal evidence of the difference between binary and continuous set of responses. We found that the type of answer is important and could yield different results. In addition, how the question is worded could play an important role in explaining its results.

## Supplementary Information

Supplementary Information.

## Data Availability

The datasets generated and/or analyzed during the current study are available from the corresponding author on request.
