# Supplementary material for: Continuous and binary sets of responses differ in the field
Source: Sci Rep. 2022 Aug 23;12:14376. doi: 10.1038/s41598-022-17907-4 (PMC9399090; doi:10.1038/s41598-022-17907-4)
Supplement: Supplementary file 1 — Supplementary Information. [file 41598_2022_17907_MOESM1_ESM.pdf]

Continuous and binary sets of responses differ in the field

Noelia Rivera-Garrido[1], M.P. Ramos-Sosa[1,*], Michela Accerenzi[2], and Pablo Brañas-Garza[1]

[1]Economics, *LoyolaBehLab*, Universidad Loyola Andalucía, Spain
[2]Fundación ETEA, *LoyolaBehLab*, Universidad Loyola Andalucía, Spain
[*]mpramos@uloyola.es

Supplementary information

S1. Questionnaire in Spanish: Menstruaciones y derechos sexuales y reproductivos.

S2. Questionnaire in English: Menstruation and sexual and reproductive rights.

S3. Extension of the Delavande test.

S4. Supplementary Table S1. Descriptive statistics.

S5. Supplementary Table S2. Schools.

S6. Supplementary Table S3. Binary vs. continuous sets of responses (Table 1 adding controls).

S7. Supplementary Table S4. Binary vs. continuous sets of responses (aggregate level).

S8. Supplementary Table S5. Binary vs. continuous sets of responses (individual level).

S9. Supplementary Table S6. Binary vs. continuous sets of responses (individual level): different samples.

S10. Supplementary Table S7. Binary vs. continuous sets of responses (individual level): Table S6 removing neutral responses (equal to 5 in continuous questions).

S11. Supplementary Table S8. Binary vs. continuous sets of responses: adding controls.

S12. Supplementary Table S9. The role of question-wording: adding controls.

S13. Supplementary Table S10. The role of question-wording

S14. Figure S1. Continuous responses. Item by item.

S1. Questionnaire (Spanish): Menstruaciones y derechos sexuales y reproductivos.

Block A

Binary setting: ¿**Cree** que una niña reciba información sobre ciclo menstrual antes de su primera menstruación...

Continuous setting: ¿**Qué probable cree** que una niña reciba información sobre ciclo menstrual antes de su primera menstruación...

SQ1- ...en la escuela?

SQ2- ...en la casa (padre, madre u otro familiar)?

SQ3- ...en la iglesia?

SQ4- ...entre sus amistades?

SQ5- ...no reciba ninguna información?

Block B

Binary setting: ¿**Cree** que cuando una niña tenga su primera menstruación...

Continuous setting: ¿**Qué probable cree** que cuando una niña tenga su primera menstruación...

SQ1- ...le digan que se ha convertido de niña a mujer?

SQ2- ...le digan que ahora debe actuar como mujer?

SQ3- ...le digan que ahora debe cuidarse de los hombres?

SQ4- ...le digan que tiene que limitar sus actividades cuando tenga la menstruación?

SQ5- ...no le dirán nada?

Block C

Binary setting: ¿**Cree** que una niña/adolescente...

Continuous setting: ¿**Qué probable cree** que una niña/adolescente...

SQ1- ...tenga fácilmente acceso a comprar productos menstruales desechables (toallas higiénicas y tampones)?

SQ2- ...tenga fácilmente acceso a comprar productos menstruales reusables (toallas de tela, copas menstruales, esponjas, otros)?

SQ3- ...tenga fácilmente acceso a productos menstruales caseros (usa telas u otros)?

SQ4- ...tenga fácilmente acceso a medicamentos para dolor menstrual?

SQ5- ...no tendrá acceso (a lo anteriormente mencionado)?

Block D

Binary setting: ¿**Cree** que una niña/adolescente, cuando tiene su menstruación, falte a clase uno o más días...

Continuous setting: ¿**Qué probable cree** que una niña/adolescente, cuando tiene su menstruación, falte a clase uno o más días...

SQ1- ...porque no tiene acceso a productos menstruales?

SQ2- ...por vergüenza (miedo a mancharse el uniforme, a que los compañeros se burlen, a que le digan algo)?

SQ3- ...porque tiene mucho dolor?

SQ4- ...porque en la escuela/colegio no hay baños/letrinas separada por sexo y/o con suficiente privacidad?

SQ5- ...porque su familia no le permite ir a la escuela/colegio?

Block E

Binary setting: ¿**Cree** que una niña entre 10 y 14 años...

Continuous setting: ¿**Qué probable cree** que una niña entre 10 y 14 años...

SQ1- ...reciba información sobre salud sexual y reproductiva en la escuela?

SQ2- ...reciba información sobre salud sexual y reproductiva en la casa (padre, madre u otro familiar)?
SQ3- ...reciba información sobre salud sexual y reproductiva en la iglesia?
SQ4- ...reciba información sobre salud sexual y reproductiva entre sus amistades?
SQ5- ...no reciba ninguna información sobre salud sexual y reproductiva?

Block F
Binary setting: ¿**Cree** que una menor de edad (menos de 18 años)...
Continuous setting: ¿**Qué probable cree** que una menor de edad (menos de 18 años)...
SQ1- ...tenga acceso a anticonceptivos hormonales auto-administrados (píldora, parche, anillo)?
SQ2- ...tenga acceso a anticonceptivos hormonales administrados por personal sanitario (inyección)?
SQ3- ...tenga acceso a preservativos?
SQ4- ...tenga acceso a otros anticonceptivos no hormonales (diafragma, DIU,. . .)?
SQ5- ...no tenga acceso a ninguno (de los mencionados anteriormente)?

Possible answers:
Binary setting: Si/No.
Continuous setting: Slider, donde 0 significa "Totalmente seguro de que no vaya a ocurrir" y 10 significa "Totalmente seguro de que vaya a ocurrir".

S2. Questionnaire: Menstruation and sexual and reproductive rights

Panel A

Binary setting: **Do you think** a girl receives menstrual cycle information before her first period...

Continuous setting: **How likely do you think** a girl will receive menstrual cycle information before...

SQ1- ...at school?

SQ2- ...at home (father, mother, or another family member)?

SQ3- ...at church?

SQ4- ...from friends?

SQ5- ...she will get no information

Panel B

Binary setting: **Do you think** that when a girl has her first period, she will be told...

Continuous setting: **How likely do you think** that when a girl has her first period, she will be told...

SQ1-...that she has become a woman?

SQ2- ..that she now has to act like a woman?

SQ3- ...that she now must be careful with men?

SQ4- ...that she has to restrict her activities when she has her period?

SQ5- ...that she will not be told anything?

Panel C

Binary setting: **Do you think** a girl/teen...

Continuous setting: **How likely do you think** a girl/teen...

SQ1- ...will have easy access to buy disposable menstrual products (pads and tampons)?

SQ2- ...will have easy access to buy reusable menstrual products (pads, menstrual cups, sponges, etc.)?

SQ3- ...will have easy access to homemade menstrual products (using a cloth or another)?

SQ4- ...will have easy access to medication for menstrual pain?

SQ5- ...will not have access to any of the above?

Panel D

Binary setting: **Do you think** when a girl/teen has her period, she will miss one or more days of school because...

Continuous setting: **How likely do you think** when a girl/teen, has her period she, will miss one or more days of school because...

SQ1- ...she does not have access to menstrual products?

SQ2- ...out of shame (fear of staining her uniform, of being teased, of being told something)?

SQ3- ...she is in a lot of pain?

SQ4- ...there are no bathrooms/ latrines in the school separated by sex and/or with enough privacy?

SQ5- ... her family does not allow her to go to school?

Panel E

Binary setting: **Do you think** a girl aged 10-14 receives...

Continuous setting: **How likely do you think** a girl aged 10-14 will receive...

SQ1- ...sexual and reproductive health information at school?

SQ2- ...sexual and reproductive health information at home (parent or another family member)?

SQ3- ...sexual and reproductive health information at church?

SQ4- ...sexual and reproductive health information from her friends?
SQ5- ...no information on sexual and reproductive health?

Panel F
Binary setting: **Do you think** a minor (under 18) will...
Continuous setting: **How likely do you think** a minor (under 18) will...
SQ1- ...have access to self-administered hormonal contraceptives (pill, patch, ring)?
SQ2- ...have access to hormonal contraceptives administered by health care providers (injection)?
SQ3- ...have access to condoms?
SQ4- ...have access to other non-hormonal contraceptives (diaphragm, IUD, etc)?
SQ5- ...not have access to any of the above?

Possible answers:
Binary setting: Yes/No
Continuous setting: Slider, where 0 is “completely sure it will not happen" and 10 is “completely sure it will happen”.

S3. Extension of the Delavande test.

To test cognitive abilities, we use an extension of the Delavande test[1] developed by[2].

Participants had to answer two groups of questions. The first group consisted of the following two questions:

Q1: *"Imagine I have a basket containing 5 apples: 1 green apple and 4 red apples. If I ask you to choose one of the apples without looking inside the basket, how likely do you think you will choose the green apple?"*

Q2: *"Imagine I have a basket containing 10 apples: 1 green apple and 9 red apples. If I ask you to choose one of the apples without looking inside the basket, how likely do you think you will choose the green apple?"*

In the second group of questions, we asked the respondents to indicate their probability of eating chicken:

Q3: "*How likely do you think you will eat chicken in the next two days (including today)?*"

Q4: *"How likely do you think you will eat chicken in the next two weeks (including today)?"*

According to Estepa et al.'s (2020) extension of the Delavande test, expectations are consistent if the following assumptions are fulfilled:

a: $Q2 < Q1$

b: $Q4 > Q3$

If an individual fails *a*, *b* or both he/she is labeled as a *low-ability subject*.

Supplementary Table S1: Descriptive Statistics.

**Variable**	**All (Mean)**	**All (SD)**	**Bin. (Mean)**	**Cont. (Mean)**	**Diff (B-C)**
Number of ones (“Yes”)	17.66	4.42	18.77	16.45	2.317***
Response time (min.)	5.11	2.41	4.07	6.23	-2.159***
Female	0.87	0.34	0.91	0.82	0.096***
Age	34.34	9.75	33.90	34.82	-0.920
Sufficient income	0.77	0.42	0.74	0.80	-0.054
Low-ability	0.29	0.46	0.29	0.30	-0.014
Having a daughter	0.65	0.48	0.66	0.63	0.030
Questions order	0.48	0.50	0.48	0.48	-0.001
**Ethnicity**					
None	0.70	0.46	0.68	0.72	-0.042
Chorti	0.18	0.39	0.20	0.17	0.035
Lenca	0.07	0.26	0.07	0.08	-0.018
Other	0.04	0.20	0.05	0.03	0.025
**Education**					
Less than primary	0.06	0.23	0.07	0.05	0.018
Primary	0.42	0.50	0.45	0.40	0.043
Secondary	0.35	0.48	0.29	0.40	-0.1**
Upper secondary	0.09	0.28	0.09	0.09	-0.002
Tertiary	0.08	0.28	0.11	0.06	0.049*
Observations	353	353	184	169	353

Note: Number of ones ("Yes"): Number of "Yes" responses by a subject in the questionnaire. Response time(min): duration of the questionnaire (in minutes). Female: dummy equal to one if female. Age: respondents’ age. Sufficient income: dummy equal to one if sufficient income for food. Low-ability: dummy equal to one if fail the Delavande test. Having a daughter: dummy equal to one if have a daughter. Question order: dummy equal to one if the participant started with blocks A, B, C. Ethnicity: (a) None: dummy equal to one if ethnicity=none; (b) Chorti: dummy equal to one if ethnicity=chorti; (c) Lenca: dummy equal to one if ethnicity=lenca; (d) Other: dummy equal to one if ethnicity=other. Education: (a) Less than primary: dummy equal to one if less than primary education ; (b) Primary: dummy equal to one if primary education; (c) Secondary: dummy equal to one if secondary education; (d) Upper secondary: dummy equal to one if upper secondary education; (e) Tertiary: dummy equal to one if tertiary education. Robust standard errors in parentheses. ***p<0.01, **p<0.05, *p<0.1.

Supplementary Table S2. Schools

School	All	Binary	Continuous	Diff
Divina Providencia	0.03	0.02	0.04	-0.02
	(0.17)	(0.15)	(0.20)	(0.02)
Jerónimo J. Reina	0.14	0.16	0.13	0.03
	(0.35)	(0.37)	(0.34)	(0.04)
Jesús Banegas Membreño	0.13	0.13	0.13	-0.005
	(0.31)	(0.33)	(0.34)	(0.03)
Jorge Portillo	0.03	0.04	0.02	0.03
	(0.17)	(0.20)	(0.13)	(0.02)
José Cecilio del Valle	0.11	0.10	0.12	-0.02
	(0.32)	(0.31)	(0.33)	(0.03)
José Maria Medina	0.11	0.11	0.10	0.01
	(0.31)	(0.32)	(0.30)	(0.03)
Los Ángeles	0.11	0.13	0.09	0.04
	(0.32)	(0.34)	(0.29)	(0.03)
Manuel Bonilla	0.10	0.09	0.11	-0.02
	(0.30)	(0.29)	(0.32)	(0.03)
San Antonio	0.11	0.08	0.14	-0.05
	(0.31)	(0.27)	(0.34)	(0.03)
San José	0.02	0.01	0.02	-0.01
	(0.13)	(0.10)	(0.15)	(0.01)
Santo Domingo Savio	0.10	0.12	0.09	0.03
	(0.31)	(0.33)	(0.29)	(0.03)
Obs.	353	184	169	353

Note: Robust standard errors in parentheses. ***p<0.01, **p<0.05, *p<0.1.

Supplementary Table S3. Binary vs. continuous set of responses (Table 1 adding controls).

Outcome:	Table 1*	Model (2)	Model (3)	Table 1*	Model (2)	Model (3)
	Aggregate Number of "Yes"			Time (min)		
**Sample: All**						
Binary	2.317***	2.333***	2.297***	-2.159***	-2.127***	-2.120***
	(0.459)	(0.458)	(0.284)	(0.238)	(0.230)	(0.169)
Dep. Var (Mean)	17.66	17.66	17.66	5.11	5.11	5.11
Observations	353	353	353	338	338	338
R-squared	0.069	0.076	0.131	0.201	0.217	0.291
**Sample: Women**						
Binary	2.253***	2.255***	2.166***	-1.991***	-2.007***	-2.030***
	(0.486)	(0.488)	(0.383)	(0.225)	(0.226)	(0.276)
Dep. Var (Mean)	17.72	17.72	17.72	4.97	4.97	4.97
Observations	306	306	306	295	295	295
R-squared	0.068	0.075	0.150	0.218	0.224	0.319
**Sample: Intermediate/ High-Ability**						
Binary	2.534***	2.569***	2.488***	-2.486***	-2.407***	-2.352***
	(0.544)	(0.551)	(0.327)	(0.291)	(0.278)	(0.180)
Dep. Var (Mean)	17.56	17.56	17.56	5.18	5.18	5.18
Observations	249	249	249	242	242	242
R-squared	0.082	0.088	0.169	0.243	0.267	0.354

Note: Table 1* replicates the results shown in the previous Table 1. The dependent variable is a dummy equal to one if the respondent agrees with the survey question. The table reports the estimated coefficients for a dummy variable equal to one if the set of responses is binary. Model (2) includes controls for age, gender, and ethnicity. Model (3) includes controls for age, gender, ethnicity, school, sufficient income, having a daughter, education, and the order of the questions in the survey.
Robust standard error in parentheses. Standard errors clustered at the school level.
***p<0.01, **p<0.05, *p<0.1.

Supplementary Table S4. Binary vs. continuous set of responses (aggregate level).

	Aggregate Number of "Yes" responses			Time (min)		
Binary	2.317***	2.772**	1.826**	-2.159***	-2.921***	-1.358**
	(0.322)	(1.010)	(0.727)	(0.155)	(0.787)	(0.485)
Binary*Female		-0.520			0.931	
		(1.193)			(0.983)	
Binary*High_ability			0.708			-1.129*
			(0.851)			(0.563)
Dep Var (mean)	17.66	17.72	17.56	5.11	4.97	5.18
Observations	353	353	353	338	338	338
R-squared	0.069	0.069	0.074	0.201	0.214	0.216

Note: The dependent variable is a dummy equal to one if the respondent agrees with the survey question. The table reports the estimated coefficients for a dummy variable equal to one if the set of responses is binary. Columns (2) and (4) include a dummy variable to account for the effect of being women and the interaction between women and binary. Columns (3) and (6) add a dummy variable to account for the effect of being intermediate/high-ability and the interaction between intermediate/high-ability and binary.
Robust standard error in parentheses. Standard errors clustered at the school level.
***p<0.01, **p<0.05, *p<0.1.

Supplementary Table S5. Binary vs. continuous set of responses (individual level).

	Coef.	SE	R-squared	Obs.
**PANEL A**				
SQ1	0.00408	(0.0482)	0.000	353
SQ2	0.0802*	(0.0403)	0.013	353
SQ3	0.0515	(0.0630)	0.003	353
SQ4	0.0607	(0.0420)	0.005	353
SQ5	0.162***	(0.0429)	0.027	353
**PANEL B**				
SQ1	-0.0481	(0.0506)	0.002	353
SQ2	-0.101*	(0.0539)	0.011	353
SQ3	0.0256	(0.0186)	0.005	353
SQ4	-0.0232	(0.0548)	0.001	353
SQ5	0.147**	(0.0593)	0.022	353
**PANEL C**				
SQ1	-0.0239	(0.0330)	0.001	353
SQ2	0.0977*	(0.0511)	0.013	353
SQ3	0.166***$^{(B)}$	(0.0302)	0.032	353
SQ4	0.0640	(0.0446)	0.013	353
SQ5	0.170***$^{(B)}$	(0.0429)	0.030	353
**PANEL D**				
SQ1	0.268***$^{(B)}$	(0.0426)	0.072	353
SQ2	0.148***	(0.0458)	0.023	353
SQ3	0.227***$^{(B)}$	(0.0293)	0.113	353
SQ4	0.208***$^{(B)}$	(0.0371)	0.044	353
SQ5	0.0183	(0.0355)	0.000	353
**PANEL E**				
SQ1	-0.00412	(0.0323)	0.000	353
SQ2	0.0479	(0.0540)	0.003	353
SQ3	0.107*	(0.0560)	0.013	353
SQ4	0.124**	(0.0407)	0.020	353
SQ5	0.136*	(0.0632)	0.019	353
**PANEL F**				
SQ1	0.0159	(0.0535)	0.000	353
SQ2	0.0159	(0.0535)	0.000	353
SQ3	0.0163	(0.0448)	0.000	353
SQ4	0.0400	(0.0505)	0.002	353
SQ5	0.148**	(0.0508)	0.023	353

Note: The dependent variable is a dummy equal to one if the respondent agrees with the survey question. The table reports the estimated coefficients for a dummy variable equal to one if the set of responses is binary. Robust standard errors in parentheses. Standard errors clustered at the school level. (B) refers to Statistically significant coefficients using Bonferroni correction.
***p<0.01, **p<0.05, *p<0.1.

Supplementary Table S6. Binary vs. continuous set of responses (individual level): different samples.

	**All**		**Women**		**Intermediate/high-Ability**	
	**Coef.**	**SE**	**Coef.**	**SE**	**Coef.**	**SE**
**PANEL A**						
SQ1	0.00408	(0.0482)	0.0106	(0.0370)	0.00323	(0.0554)
SQ2	0.0802*	(0.0403)	0.106**	(0.0405)	0.0864**	(0.0336)
SQ3	0.0515	(0.0630)	0.0805	(0.0578)	0.0901	(0.0672)
SQ4	0.0607	(0.0420)	0.0947	(0.0534)	0.0600	(0.0534)
SQ5	0.162***	(0.0429)	0.124**	(0.0451)	0.174***	(0.0429)
**PANEL B**						
SQ1	-0.0481	(0.0506)	-0.0740	(0.0593)	-0.0224	(0.0594)
SQ2	-0.101*	(0.0539)	-0.110*	(0.0561)	-0.0794	(0.0546)
SQ3	0.0256	(0.0186)	0.0184	(0.0175)	0.0195	(0.0165)
SQ4	-0.0232	(0.0548)	-0.0347	(0.0447)	-0.0290	(0.0528)
SQ5	0.147**	(0.0593)	0.130*	(0.0643)	0.154**	(0.0675)
**PANEL C**						
SQ1	-0.0239	(0.0330)	-0.0295	(0.0291)	-0.0204	(0.0451)
SQ2	0.0977*	(0.0511)	0.0815*	(0.0447)	0.0562	(0.0480)
SQ3	0.166***	(0.0302)	0.166***	(0.0340)	0.231***	(0.0363)
SQ4	0.0640	(0.0446)	0.0624	(0.0424)	0.0822	(0.0495)
SQ5	0.170***	(0.0429)	0.159***	(0.0448)	0.178***	(0.0547)
**PANEL D**						
SQ1	0.268***	(0.0426)	0.247***	(0.0695)	0.288***	(0.0594)
SQ2	0.148***	(0.0458)	0.117*	(0.0571)	0.166**	(0.0717)
SQ3	0.227***	(0.0293)	0.223***	(0.0426)	0.231***	(0.0351)
SQ4	0.208***	(0.0371)	0.186***	(0.0321)	0.207***	(0.0445)
SQ5	0.0183	(0.0355)	0.0109	(0.0285)	0.0163	(0.0407)
**PANEL E**						
SQ1	-0.00412	(0.0323)	0.0155	(0.0337)	-0.00770	(0.0291)
SQ2	0.0479	(0.0540)	0.0631	(0.0528)	0.0956*	(0.0454)
SQ3	0.107*	(0.0560)	0.125*	(0.0577)	0.160**	(0.0642)
SQ4	0.124**	(0.0407)	0.151***	(0.0408)	0.138*	(0.0631)
SQ5	0.136*	(0.0632)	0.137*	(0.0666)	0.177**	(0.0662)
**PANEL F**						
SQ1	-0.0166	(0.0395)	0.0109	(0.0456)	-0.0325	(0.0501)
SQ2	0.0159	(0.0535)	0.00673	(0.0527)	-0.0292	(0.0734)
SQ3	0.0163	(0.0448)	0.0220	(0.0517)	-0.0468	(0.0523)
SQ4	0.0400	(0.0505)	0.0194	(0.0631)	0.0189	(0.0608)
SQ5	0.148**	(0.0508)	0.134*	(0.0703)	0.169**	(0.0556)
Obs.	353		306		249	

Note: The dependent variable is a dummy equal to one if the respondent agrees with the survey question. The table reports the estimated coefficients for a dummy variable equal to one if the set of responses is binary. Robust standard errors in parentheses. Standard errors clustered at the school level.
***p<0.01, **p<0.05, *p<0.1.

Supplementary Table S7. Binary vs. continuous set of responses (individual level): Table S6 removes neutral responses (equal to 5 in continuous questions).

	All		Women		Intermediate/high-Ability	
	**Coef.**	**SE**	**Coef.**	**SE**	**Coef.**	**SE**
**PANEL A**						
SQ1	-0.00862	(0.0509)	0.000444	(0.0386)	-0.0151	(0.0579)
SQ2	0.0251	(0.0398)	0.0368	(0.0304)	0.0272	(0.0292)
SQ3	0.0952	(0.0571)	0.125**	(0.0551)	0.122*	(0.0579)
SQ4	0.0237	(0.0391)	0.0585	(0.0582)	0.0134	(0.0582)
SQ5	0.172***	(0.0447)	0.135**	(0.0504)	0.197***	(0.0388)
**PANEL B**						
SQ1	-0.0644	(0.0494)	-0.0936	(0.0562)	-0.0418	(0.0610)
SQ2	-0.107*	(0.0544)	-0.120*	(0.0600)	-0.0776	(0.0582)
SQ3	0.00894	(0.0172)	0.00453	(0.0181)	-0.00504	(0.0186)
SQ4	-0.0481	(0.0621)	-0.0595	(0.0513)	-0.0558	(0.0627)
SQ5	0.177**	(0.0642)	0.160**	(0.0699)	0.200**	(0.0758)
**PANEL C**						
SQ1	-0.0559*	(0.0291)	-0.0623**	(0.0272)	-0.0507	(0.0423)
SQ2	0.103*	(0.0519)	0.0902*	(0.0451)	0.0560	(0.0522)
SQ3	0.175***	(0.0319)	0.177***	(0.0382)	0.241***	(0.0395)
SQ4	0.0400	(0.0412)	0.0324	(0.0366)	0.0475	(0.0461)
SQ5	0.179***	(0.0436)	0.173***	(0.0458)	0.189***	(0.0533)
**PANEL D**						
SQ1	0.287***	(0.0461)	0.260***	(0.0746)	0.307***	(0.0649)
SQ2	0.127**	(0.0441)	0.0881	(0.0592)	0.144*	(0.0730)
SQ3	0.192***	(0.0258)	0.188***	(0.0387)	0.194***	(0.0329)
SQ4	0.227***	(0.0404)	0.204***	(0.0355)	0.232***	(0.0504)
SQ5	0.0277	(0.0387)	0.0187	(0.0334)	0.0261	(0.0419)
**PANEL E**						
SQ1	-0.0199	(0.0289)	-0.00376	(0.0302)	-0.0226	(0.0210)
SQ2	-0.00104	(0.0528)	0.00280	(0.0495)	0.0422	(0.0347)
SQ3	0.113*	(0.0620)	0.141*	(0.0653)	0.153**	(0.0647)
SQ4	0.100**	(0.0432)	0.124**	(0.0405)	0.103	(0.0670)
SQ5	0.139**	(0.0602)	0.139*	(0.0656)	0.173**	(0.0619)
**PANEL F**						
SQ1	-0.0324	(0.0396)	-0.00397	(0.0473)	-0.0451	(0.0495)
SQ2	0.0284	(0.0577)	0.0160	(0.0551)	-0.0313	(0.0756)
SQ3	-0.00641	(0.0436)	-0.00595	(0.0523)	-0.0618	(0.0432)
SQ4	0.0674	(0.0493)	0.0580	(0.0585)	0.0378	(0.0657)
SQ5	0.155**	(0.0514)	0.148*	(0.0715)	0.189***	(0.0553)
Obs.	353		306		249	

Note: The dependent variable is a dummy equal to one if the respondent agrees with the survey question. The table reports the estimated coefficients for a dummy variable equal to one if the set of responses is binary. Robust standard error clustered by school in parentheses. Standard errors clustered at the school level.
***p<0.01, **p<0.05, *p<0.1.

Supplementary Table S8. Binary vs. continuous set of responses: adding controls.

	Table S8*		Model 2		Model 3	
	**Coef.**	**SE**	**Coef.**	**SE**	**Coef.**	**SE**
**PANEL A**						
SQ1	0.00408	(0.0482)	-0.0106	(0.0509)	-0.0179	(0.0502)
SQ2	0.0802*	(0.0403)	0.0779*	(0.0383)	0.0756*	(0.0399)
SQ3	0.0515	(0.0630)	0.0668	(0.0649)	0.0748	(0.0635)
SQ4	0.0607	(0.0420)	0.0658	(0.0351)	0.0388	(0.0351)
SQ5	0.162***	(0.0429)	0.169***	(0.0419)	0.203***	(0.0435)
**PANEL B**						
SQ1	-0.0481	(0.0506)	-0.0581	(0.0583)	-0.0833	(0.0534)
SQ2	-0.101*	(0.0539)	-0.103*	(0.0517)	-0.113*	(0.0587)
SQ3	0.0256	(0.0186)	0.0190	(0.0163)	0.0232	(0.0146)
SQ4	-0.0232	(0.0548)	-0.0223	(0.0547)	-0.0398	(0.0583)
SQ5	0.147**	(0.0593)	0.152**	(0.0564)	0.168**	(0.0580)
**PANEL C**						
SQ1	-0.0239	(0.0330)	-0.0310	(0.0306)	-0.0413	(0.0317)
SQ2	0.0977*	(0.0511)	0.105*	(0.0476)	0.0882	(0.0579)
SQ3	0.166***	(0.0302)	0.151***	(0.0258)	0.130***	(0.0298)
SQ4	0.0640	(0.0446)	0.0606	(0.0464)	0.0566	(0.0466)
SQ5	0.170***	(0.0429)	0.181***	(0.0400)	0.202***	(0.0442)
**PANEL D**						
SQ1	0.268***	(0.0426)	0.270***	(0.0499)	0.256***	(0.0552)
SQ2	0.148***	(0.0458)	0.130**	(0.0529)	0.108	(0.0605)
SQ3	0.227***	(0.0293)	0.220***	(0.0314)	0.210***	(0.0311)
SQ4	0.208***	(0.0371)	0.207***	(0.0400)	0.213***	(0.0415)
SQ5	0.0183	(0.0355)	0.0245	(0.0293)	0.0246	(0.0336)
**PANEL E**						
SQ1	-0.00412	(0.0323)	-0.0122	(0.0291)	-0.0102	(0.0263)
SQ2	0.0479	(0.0540)	0.0337	(0.0489)	0.0484	(0.0525)
SQ3	0.107*	(0.0560)	0.127**	(0.0539)	0.139**	(0.0555)
SQ4	0.124**	(0.0407)	0.102**	(0.0416)	0.0921*	(0.0429)
SQ5	0.136*	(0.0632)	0.149**	(0.0553)	0.173**	(0.0597)
**PANEL F**						
SQ1	-0.0166	(0.0395)	-0.0102	(0.0426)	-0.0135	(0.0398)
SQ2	0.0159	(0.0535)	0.0222	(0.0544)	0.0348	(0.0494)
SQ3	0.0163	(0.0448)	0.0284	(0.0460)	0.0307	(0.0488)
SQ4	0.0400	(0.0505)	0.0635	(0.0559)	0.0600	(0.0564)
SQ5	0.148**	(0.0508)	0.155**	(0.0512)	0.171***	(0.0497)
Obs.	353		306		249	

Note: Table S8* replicates the results of Table S6. The dependent variable is a dummy equal to one if the respondent agrees with the survey question. The table reports the estimated coefficients for a dummy variable equal to one if the set of responses is binary. Model (2) includes controls for age, gender, and ethnicity. Model (3) includes controls for age, gender, ethnicity, school, sufficient income, having a daughter, education, and the order of the questions in the survey.
Robust standard error clustered by school in parentheses.
***p<0.01, **p<0.05, *p<0.1.

Supplementary Table S9. The role of question-wording: adding controls.

	Negative	Prescriptive	Others
Sample: All			
Binary	1.402***	-0.129	1.024***
	(0.244)	(0.8)	(0.226)
Dep. Var (Mean)	3.42	2.09	12.14
Observations	353	353	353
R-squared	0.166	0.092	0.125
Sample: Women			
Binary	1.203***	-0.168	1.131***
	(0.309)	(0.0)	(0.278)
Dep. Var (Mean)	3.44	2.0	12.18
Observations	306	306	306
R-squared	0.145	0.158	0.139
Sample: Intermediate/ High-ability			
Binary	1.483***	-0.0939	1.099***
	(0.230)	(0.124)	(0.239)
Dep. Var (Mean)	3.29	2.08	12.1
Observations	249	249	249
R-squared	0.043	0.082	0.165

Note: The dependent variable is the number of "Yes" responses by the subject in the questionnaire. The "Negative" column refers to the negative questions. The "Prescriptive" column refers to the prescriptive questions. The "Others" column refers to questions that cannot be classified as negative or prescriptive. All coefficients have been estimated by controlling for age, gender, ethnicity, school, sufficient income, having a daughter, education, and the order of the questions in the survey.
Standard errors clustered by school and individual in parentheses.
*** p<0.01, ** p<0.05, p<0.1

Supplementary Table S10. The role of question-wording

Sample:	All	Women	Intermediate/ High-ability
Binary	0.0609***	0.0659***	0.0663***
	(0.0170)	(0.0183)	(0.0207)
Binary*Negative	0.0963***	0.0751**	0.104***
	(0.0297)	(0.0325)	(0.0344)
Binary*Prescriptive	-0.0937***	-0.108***	-0.0960***
	(0.0258)	(0.0278)	(0.0306)
Dep. Var (Mean)	0.52	0.55	0.53
Observations	10,590	9,180	7,470
R-squared	0.049	0.049	0.056

Note: The dependent variable is a dummy equal to one if the respondent agrees with the survey question. "Negative" refers to negative questions. "Prescriptive" refers to prescriptive questions. The comparison groups if "others" questions that cannot be classified as negative or prescriptive.
Standard errors clustered by school in parentheses.
*** p<0.01, ** p<0.05, p<0.1

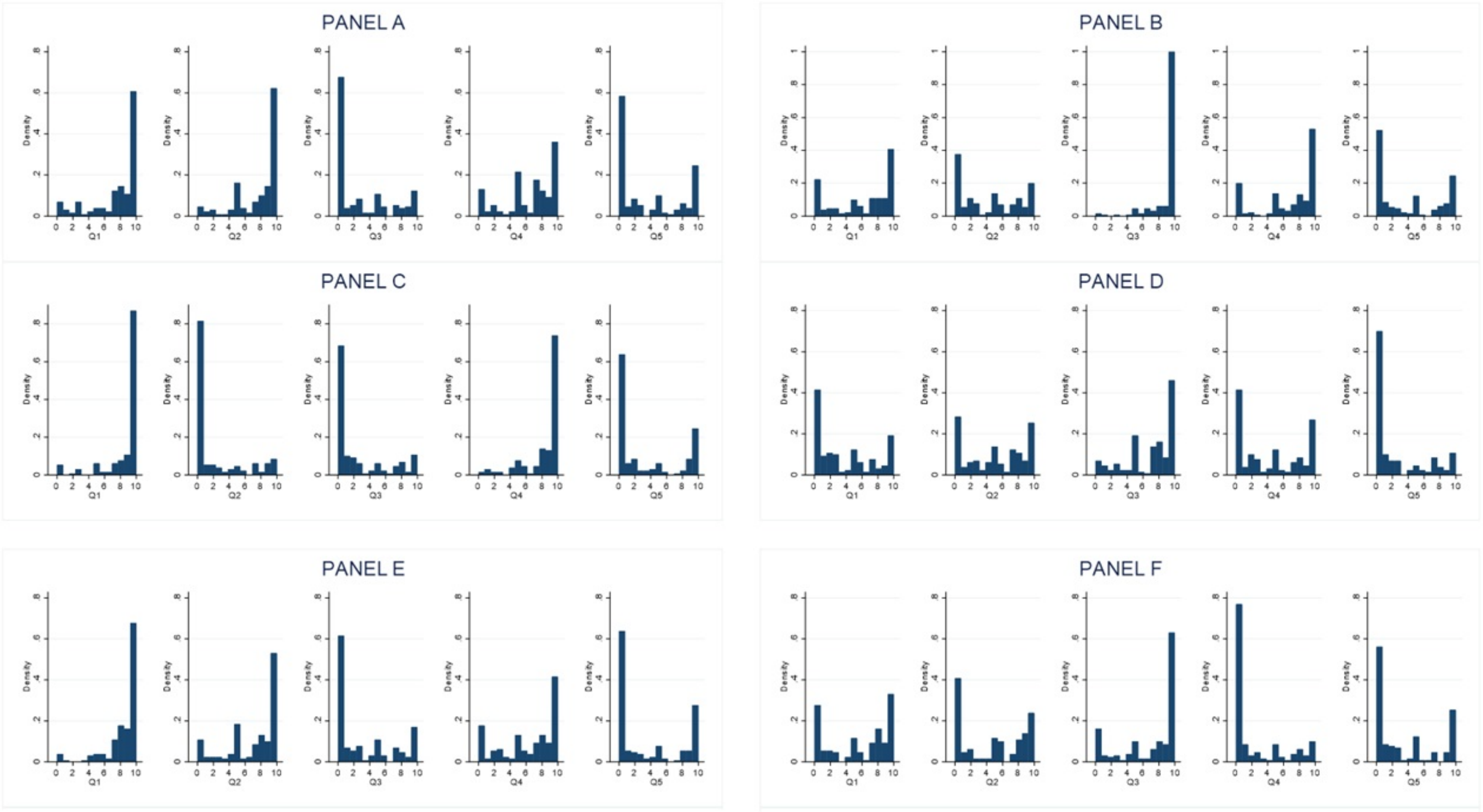

Figure S1. Continuous answers. Item by item.

References

1. Delavande, A. & Kohler, H.-P. Subjective expectations in the context of hiv/aids in malawi. *Demogr. research* **20**, 817 (2009).
2. Estepa, L., Jorrat, D., Orozco, V. & Rascón-Ramirez, E. Beyond beans: Eliciting probabilities using slider in the field. *Mimeo* (2020).
